# Over-Expression of CYP2E1 mRNA and Protein: Implications of Xenobiotic Induced Damage in Patients with *De Novo* Acute Myeloid Leukemia with inv(16)(p13.1q22); *CBFβ-MYH11*

**DOI:** 10.3390/ijerph9082788

**Published:** 2012-08-03

**Authors:** Rashmi Kanagal-Shamanna, Weiqiang Zhao, Saroj Vadhan-Raj, Martin H. Nguyen, Michael H. Fernandez, L. Jeffrey Medeiros, Carlos E. Bueso-Ramos

**Affiliations:** 1 Department of Hematopathology, The University of Texas MD Anderson Cancer Center, 1515 Holcombe Blvd., Unit 0072, Houston, TX 77030, USA; Email: Rkanagal@mdanderson.org (R.K.-S.); mhnguyen@mdanderson.org (M.H.N.); mhfernan@mdanderson.org (M.H.F.); ljmedeiros@mdanderson.org (L.J.M.); 2 Department of Pathology, Ohio State University Medical Center, Columbus, OH 43210, USA; 3 Department of Sarcoma Medical Oncology, The University of Texas MD Anderson Cancer Center, 1515 Holcombe Blvd., Unit 0072, Houston, TX 77030, USA; Email: svadhanr@mdanderson.org

**Keywords:** CYP2E1, NQO1, AML with inv(16), *de novo* AML, CBFB-MYH11, benzene, xenobiotic induced damage

## Abstract

Environmental exposure to benzene occurs through cigarette smoke, unleaded gasoline and certain types of plastic. Benzene is converted to hematotoxic metabolites by the hepatic phase-I enzyme CYP2E1, and these metabolites are detoxified by the phase-II enzyme NQO1. The genes encoding these enzymes are highly polymorphic and studies of these polymorphisms have shown different pathogenic and prognostic features in various hematological malignancies. The potential role of different cytochrome p450 metabolizing enzymes in the pathogenesis of acute myeloid leukemia (AML) in an area of active interest. In this study, we demonstrate aberrant CYP2E1 mRNA over-expression by quantitative real-time polymerase chain reaction in 11 cases of *de novo* AML with inv(16); CBFβ-MYH11. CYP2E1 mRNA levels correlated with *CBFβ-MYH11* transcript levels and with bone marrow blast counts in all cases. CYP2E1 over-expression correlated positively with NQO1 mRNA levels (R^2^ = 0.934, n = 7). By immunohistochemistry, CYP2E1 protein was more frequently expressed in AML with inv(16) compared with other types of AML (*p* < 0.001). We obtained serial bone marrow samples from two patients with AML with inv(16) before and after treatment. CYP2E1 mRNA expression levels decreased in parallel with *CBFβ-MYH11* transcript levels and blast counts following chemotherapy. In contrast, CYP1A2 transcript levels did not change in either patient. This is the first study to demonstrate concurrent over-expression of CYP2E1 and NQO1 mRNA in AML with inv(16). These findings also suggest that a balance between CYP2E1 and NQO1 may be important in the pathogenesis of AML with inv(16).

## 1. Introduction

Benzene is a long established leukemogen implicated in the pathogenesis of myelodysplastic syndromes (MDS) and acute leukemias [1]. Although benzene exposure in the workplace is minimal and regulated, exposure to small but measurable amounts of benzene still occurs, as shown in blood and urine samples of the general population. Benzene is commonly present in cigarette smoke, unleaded gasoline and certain types of plastic [2,3]. The hematotoxic effects of benzene are elicited when it is converted to its toxic metabolites (phenols) including hydroquinone, 1,2,4-benzenetriol and catechol. This conversion is mediated by the hepatic phase-I metabolizing cytochrome p450, family 2, subfamily E, polypeptide 1 enzyme, CYP2E1 [4]. These toxic metabolites are detoxified and degraded by the phase-II enzyme NAD(P)H: quinine oxidoreductase 1 (NQO1) enzyme [5]. Thus, it is reasonable to hypothesize that elevated CYP2E1 activity and/or decreased activity of NQO1 predisposes individuals exposed to xenobiotics to a greater risk of leukemia [6]. 

*CYP2E1* is a hepatic monooxygenase involved in xenobiotic metabolism. The *CYP2E1* gene is associated with the metabolism of many carcinogens. Polymorphisms of CYP2E1 and their possible effects have been reported by others. The CYP2E1*5 allele is associated with a higher risk of *de novo* acute myeloid leukemia (AML) and acute lymphoblastic leukemia [7]. *In-vitro* experiments have shown that lymphocytes with the CYP2E1 single nucleotide polymorphisms rs2070673TT and rs2030920CC induce greater transcription and enzyme activity and experience greater damage to cellular DNA when exposed to phenol [8]. The CYP2E1*5B(C-1019T) polymorphism has not been found to be associated with therapy-related AML or MDS [9]. The CYP2E1*07 (rs2070673) allele has been shown to be associated with increased survival rate in patients with chronic lymphocytic leukemia/small lymphocytic lymphoma (CLL/SLL) [10]. Hence, different allelic expressions of the CYP2E1 gene have different pathogenic implications and prognostic features in various hematological malignancies. Furthermore, the phenotypic functional activity of CYP2E1 does not necessarily correlate with the presence of polymorphisms, and overall functional assessment is more accurate than assessing for polymorphisms. Increased expression of CYP2E1 has been reported to be associated with liver diseases, including alcoholic hepatitis and non-alcoholic steatohepatitis, and is thought to be involved in the pathogenesis of these diseases [11,12]. However, in most studies, there has been little correlation of CYP2E1 mRNA levels with either morphologic findings or immunohistochemical data in AML. 

NQO1 is a phase-II enzyme responsible for detoxification of toxic metabolites of benzene. Low NQO1 activity, implied by the presence of *NQO1* gene C609T polymorphism, has been shown in therapy-related myeloid neoplasms [13], benzene exposure associated leukopenias [6,14], infant leukemias associated with *MLL* gene rearrangements [15] and a subset of *de novo* AML [5]. These findings support the concept that normal environmental agents that are detoxified by NQO1 are risk factors for acute leukemias [5]. In a study by Smith and colleagues focused on *de novo* AML, the strongest association of the NQO1 polymorphism was with AML with inv(16)*/CBFβ-MYH11* [5]. In addition, Smith *et al*., showed that the polymorphism on *NQO1* was validated at the protein and activity level both *in-vitro* and *in-vivo*. While it has been hypothesized that the *NQO1* gene, located on chromosome 16q22.1 may be disrupted by the translocation [5], it is also possible that myeloid cells with this cytogenetic abnormality are particularly susceptible to these environmental toxins. Furthermore, in a gene expression profiling study of AML with inv(16)*/CBFβ-MYH11*, we observed that *CYP2E1* was one of the four most differentially expressed genes, upregulated 3.3-fold times in AML with inv(16) [16]. 

AML with inv(16) carries a favorable prognosis [17,18] despite over-expression of CYP2E1. We describe the mRNA levels and protein expression pattern of CYP2E1 in *de novo* AML with inv(16) and correlate CYP2E1 expression with blast counts and *CBFβ-MYH11* chimeric transcript levels. We also assessed the status of the NQO1 mRNA levels. 

## 2. Materials and Methods

### 2.1. Case Selection

This study was approved by the Institutional Review Board of The University of Texas MD Anderson Cancer Center. We prospectively obtained fresh bone marrow (BM) samples from 11 patients with AML with inv(16), two patients with acute myelomonocytic leukemia (AMML) without inv(16), one patient with a myeloproliferative neoplasm (MPN), one patient with hairy cell leukemia (HCL), and one patient with CLL/SLL for gene expression profiling by real-time polymerase chain reaction. We also performed immunohistochemical studies by collecting 63 bone marrow (BM) biopsy specimens that were formalin-fixed and paraffin-embedded from the archives. These included 27 cases of AML with inv(16), 10 cases of AMML, 22 cases of other types of AML, two cases of chronic myelomonocytic leukemia, and two negative and apparently normal BM specimens. Clinical, pathological and molecular results were obtained from the medical records. 

### 2.2. Conventional Cytogenetic Analysis

Conventional cytogenetic analysis was performed on BM aspirate samples using standard techniques described previously [19]. At least 20 metaphases were analyzed. The International System for Human Cytogenetic Nomenclature was used for reporting the results [20]. 

### 2.3. Real-Time Quantitative Reverse Transcriptase Polymerase Chain Reaction (qRT-PCR) of CBFβ-MYH11 Transcripts, CYP2E1, CYP1A2 and NQO1

RNA was isolated from fresh BM aspirate specimens using the TRIzol^®^ Plus RNA Purification Kit according to the manufacturer’s protocol (Invitrogen, Grand Island, NY, USA). cDNA synthesis was performed using the QuantiTect RT Kit (Qiagen, Valencia, CA, USA) according to manufacturer’s instructions [14]. The commercially produced normal BM CD34 cDNA (AllCells, LLC, Emeryville, CA, USA) was also used in this study. Quantitative measurements of *CBFβ-MYH11* (Types A, C, and E) expression levels in BM samples were measured by using Taqman Universal PCR MasterMix (Applied Biosystems Inc., Carlsbad, CA, USA). Briefly, 2*–*5 µL cDNA was mixed in a total 25 µL qRT-PCR reaction mixture containing primers and probes (Ipsogen, New Brunswick, NJ, USA) in which *C-ABL* was used as the internal control, and performed in an ABI PRISM 7700 Sequence Detection System (Applied Biosystems, Foster City, CA, USA) under standardized conditions. The PCR reaction for detection of *CYP2E1, CYP1A2*, and *NQO1*, was similar to that described above but was performed in Mx3000P real time PCR (Stratagene, La Jolla, CA, USA) using a standard 2 step qPCR cycling conditions set by the instrument. The probes and forward and reverse primers in the latter reactions were synthesized by Applied Biosystems (ABI) as follows. *CYP2E1* probe (P) 5’-6FAM-(865)TGGACGGTATCACCG-MGB, Forward (F): 5’-(843)AGTGCAGAGCGCTTGTACACA, and Reverse (R): 5’-(901)AAGAACAGGTCGGCCACAGT; *CYP1A2* P: 5’-6FAM-(865) AGGCCTTCATCCTGG-MGB, F: 5’-(1130) ACCCCAGCTGCCCTACTT G-3’, and R: 5’-(1187) GAAGGAGGAGTGTCGGAAGGT; *NQO-1*: P: 5-6FAM-AGTCTTAGAACCTCAACTGACAT-MGB, F: 5-AGAGTGGCATTCTGCATTTCTG, and R: 5-CTGGAGTGTGCCCAATGCTA; *β-Actin* P: 5-6FAM-ATCAAGATCATTGCTCCTCCTGAGCGC, F: 5-CCCTGGCACCCAGCAC and R: 5-GCCGATCCACACGGAGTAC. All reactions were run twice with triplicates in each run. The ratio of copy number of target gene over copy number of internal control gene was used for expression level in each specimen. 

### 2.4. Immunohistochemical Analysis

Immunohistochemical staining for CYP2E1 was performed using 4 micron thick sections of formalin-fixed, paraffin-embedded (FFPE) BM biopsy or aspirate clot sections. Briefly, sections were placed in a 60 °C oven for 1 hour, cooled, and deparaffinized and rehydrated through xylenes and graded ethanol solutions to water. All slides were quenched for 5 minutes in 3% hydrogen peroxide for endogenous peroxidase. Antigen retrieval was performed using a heat-induced method in which the specimens were placed in a citric acid solution (pH 6.1) for 25 minutes at 94 °C and cooled for 15 minutes. Slides were then placed on Autostainer (Dako, Carpinteria, CA, USA) for immunohistochemistry. The antibody, anti-human CYP2E1 (Chemicon International, Temecula, CA, USA), was used at a dilution of 1:300 and incubated for 30 minutes at room temperature. The detection system was either Mach 4 alkaline phosphatase (Biocare Medical, Concord, CA, USA) with Vulcan Fast Red to produce a bright fushia precipitate or Envision Plus HRP (Dako) with diaminobenzidine (DAB) chromogen to produce a brown precipitate. Slides were counterstained with Richard Allen hematoxylin. Hepatic tissue was used as a control, showing intense cytoplasmic staining in normal hepatocytes. 

The immunohistochemistry slides were reviewed using an eyepiece reticle (grid). Any intensity of cytoplasmic reactivity for CYP2E1 in blasts was considered positive. Staining of the blasts was assessed in three random microscopic fields and averaged. The proportion of blasts staining positive was scored semiquantitatively as positive, focal/weakly positive, or negative. Bone marrow stromal cells and endothelial cells were negative.

### 2.5. Cell Culture, Treatment and Quantitative Real-Time RT-PCR Assay

K562 (chronic myelogenous leukemia in erythroid blast crisis) cells were cultured at 37 °C with 2% CO2 in complete culture medium (10% FBS in RPMI-1640). Twenty-four hours following plating, hydroquinone (HQ, benzene-1,4-diol or quinol, Sigma, St. Louis, MO, USA), diluted by DMSO and (BQ, 1,4-benzoquinone, Sigma-Aldrich, diluted by ethanol) were added to the plates. The cells were exposed to these chemicals at two different concentrations, 0.1 and 1.0 uM. mRNA quantification of enzymes CYP2E1, CYP1A2 and NQO1 was performed at two different time points, twenty-four and forty-eight hours after exposure to HQ and BQ. RNA was isolated from K562 cells using TRIzol^®^ Plus RNA Purification Kit according to the manufacturer’s instructions (Invitrogen). This was followed by cDNA reverse transcription using SuperScript™ II Reverse Transcriptase (Invitrogen) with 1–10 μg RNA. Quantitative RT-PCR was performed using the primers as described above. Fold changes were used for expression differences between treated and control groups, and calculated as described by Pfaffl *et al*. [21]. 

### 2.6. Statistical Analysis

The Chi square test was used to compare distinct groups. A *p*-value ≤ 0.05 was considered significant. 

## 3. Results

### 3.1. Clinical Findings

Eleven cases of AML with inv(16) were selected for gene expression profiling. The diagnosis of AML with inv(16)/*CBFβ-MYH11* was established by demonstrating inv(16)(p13q22)/*CBFβ-MYH11*. Based on the 2008 World Health Organization (WHO) classification, this type of AML is defined by the presence of recurrent balanced chromosomal rearrangement via inv(16)(p13.1q22) or t(16;16)(p13.1;q22), irrespective of the blast count. Morphologically, this type of AML shows myelomonocytic differentiation and eosinophilia with abnormal granulation. The clinicopathologic features of these cases are summarized in Table 1. There were nine men and two women, with a median age of 42.5 years (range, 10–65). All patients presented *de novo*, without prior treatment. Eight of 11 patients had ≥20% blasts. The median BM blast count at presentation was 52% (range, 1–88). Five of 11 (45%) patients had eosinophilia (>4%). None of these patients had received any known CYP2E1 inducing drugs including isoniazid and ethanol. 

**Table 1:** Clinicopathologic features of patients with AML with inv(16) for mRNA assessment.

Age/Sex	Blast %	Eo %	Log10 2E1/actin	Log10 CBFB/MYH11	F/U	Other genetic mutations	Conventional cytogenetics on BM
45/M	47	7	4.64	2.40	Alive	None	46,XY,inv(16)(p13.1q22) [1] 47,XY,inv(16)(p13.1q22),+22 [2]
40/M	88	3	4.40	2.75	Dead	*NRAS*	46,XY,inv(16)(p13.1q22) [20]
10/F	48	12	4.08	2.86	Alive	None	46,XX,inv(16)(p13.1q22) [19]/46,XX,+8,inv(16)(p13.2q22) [1]
24/M	77	6	4.66	2.41	Alive	None	46,XY,inv(16)(p13q22) [20]
36/M	73	8	4.11	3.00	Alive	*NRAS*	46,XY,inv(16)(p13q22) [15] 46,XY [5]
51/M	83	0	3.52	2.30	Alive	*FLT3*	46,XX,inv(16)(p13.1q22) [20]
65/M	56	10	3.50	2.30	Alive	None	46,XY,inv(16)(p13.1q22) [20]
35/M	62	5	3.41	2.01	Alive	*NRAS*	46,XY,inv(16)(p13.1q22) [20]
53/M	1	3	0.11	0.00	Alive	None	46,XY,inv(16)(p13.1q22) [1]/46,XY, t(6;20)(p21.3;q11.2) inv(16)(p13.1q22) [9] /46,XY [10]
26/M	1	1	0.06	0.21	Alive	None	BM, 46,XY; Soft tissue myeloid sarcoma showed inv(16) by FISH (Conventional karyotyping ND)
45/F	2	3	0.03	0.00	Alive	None	BM, 46,XY [19]; history of inv(16); current BM is in CR

Eo: Eosinophil; BM: Bone marrow; F/U: Follow-up; ND: Not done; CR: Complete remission.

Cases 1–8 with ≥20% blasts showed morphological evidence of AML in the BM aspirate samples available for testing. Case 9 had involvement by minimal residual AML, supported by the detection of inv(16) abnormality by conventional cytogenetic analysis. Cases 10 and 11 had a history of AML with inv(16) but were in complete remission as shown by the absence of *CBFβ/MYH11* transcripts in the current BM sample. 

### 3.2. Real-Time qRT-PCR Findings of CBFβ-MYH11 Transcripts, CYP2E1, CYP1A2 and NQO1

**Figure 1:**
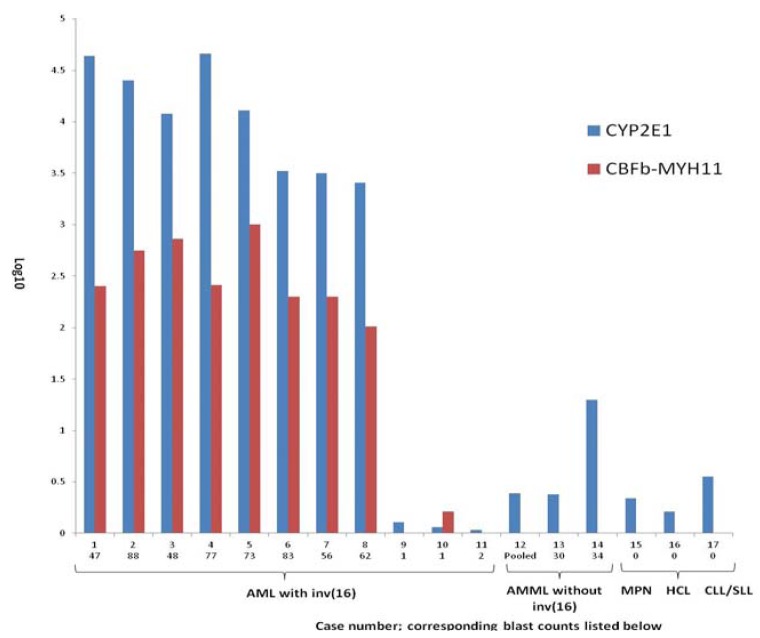
Comparison of expression levels of CYP2E1 mRNA in AML with inv(16), AML without inv(16), myeloproliferative neoplasm (MPN), hairy cell leukemia (HCL) and chronic lymphocytic leukemia/small lymphocytic lymphoma (CLL/SLL). The CYP2E1 expression levels (blue) are compared with *CBFβ-MYH11* transcript levels (maroon) in 11 cases of AML with inv(16), 2 cases of AMML without inv(16) and 1 case each of MPN, HCL and CLL/SLL. The BM blast counts are listed at the bottom of the figure. “Pooled” indicates the pooled RNA extracted from 20 BM cases of AML without inv(16).

*CYP2E1* levels correlated well with *CBFβ-MYH11* transcript levels in all cases of AML with inv(16), and were proportional to the BM blast count. As a control, we tested CYP2E1 mRNA from two cases of newly diagnosed untreated AMML without inv(16) and pooled RNA from 20 untreated AMML without inv(16). Of these, only one case of AMML showed minimal expression of CYP2E1 while the remaining cases were negative. RNA specimens from patients with other diseases including MPN, HCL and CLL/SLL cases were either negative or had minimal CYP2E1 expression (Figure 1). 

**Figure 2:**
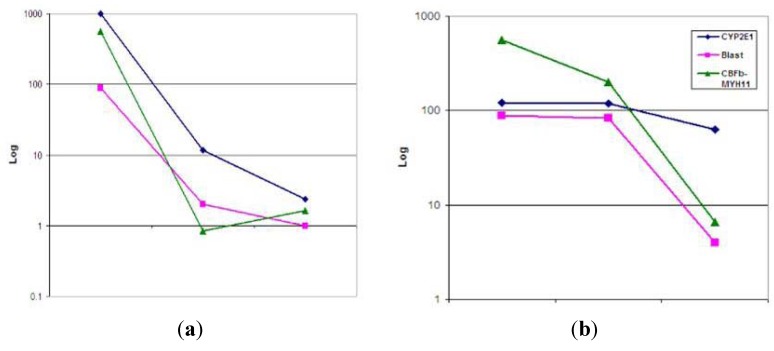
CYP2E1 mRNA expression in two different cases of AML with inv(16) (**a,b**) who were tested before (left test point) and 1 and 3 months after starting conventional chemotherapy with cytarabine consolidation.

Furthermore, in two patients with AML with inv(16), BM samples collected before and after standard chemotherapy with cytarabine were available. CYP2E1 mRNA expression was assessed before treatment, and subsequently at 1 and 3 months after starting conventional chemotherapy with cytarabine consolidation. In the first patient, the BM blast count and *CBFβ-MYH11* transcript levels dramatically decreased within the first month of therapy with a corresponding decrease in CYP2E1 by almost 3 logs. In the second case, the patient did not show a significant decrease in the blast count and *CBFβ-MYH11* transcript levels in the first month. However, with continued therapy, the patient showed a significant decrease in both of the above parameters; the patient went into complete remission at the subsequent bone marrow examination. Although the profiles of the two cases are different, in both of these cases, *CYP2E1* expression levels decreased in parallel with the CBFB-MYH11 expression levels and blast counts following treatment (Figure 2A, case 1; and Figure 2B, case 2). In comparison, CYP1A2 expression levels did not change in either patient.

In seven of the 11 unsorted BM specimens, the expression levels of CYP2E1, CYP1A2 and NQO1 from each individual were compared. Over-expression of CYP2E1 correlated positively with expression of NQO1, (*R*^2^ = 0.934, n = 7) and correlated negatively with CYP1A2 (*R*^2^ = −0.506), (Table 2).

**Table 2:** Correlation of Expression of CYP2E1, CYP1A2, and NQO1 in AML with inv(16).

Category	CYP2E1/ABL	CYP1A2/ABL *	NQO1/ABL *
Mean (n = 7)	25.32	0.77	52.11
*R* ^2^		−0.506	0.934

* *p*-*value <* 0.001.

**Figure 3:**
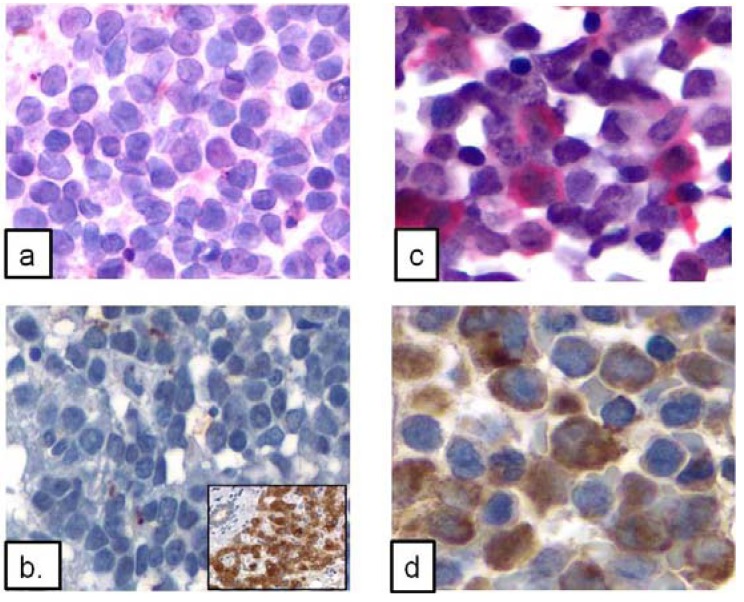
Representative CYP2E1 protein expression shown by immunohistochemistry in leukemic cells of AML with inv(16) and AML without inv(16) as a control. (**a,b**) Low level or absent CYP2E1 expression in two different cases of AML without inv(16) (hematoxylin & eosin, 400×); Inset (**b**) CYP2E1 expression in normal human liver tissue as a control; (**c,d**) Strong CYP2E1 expression in two different cases of AML with inv(16) (500×). In (**a,c**), new fuschsin was used with alkaline phosphatase; whereas in (**b,d**) DAB was used with horseradish peroxidase.

### 3.3. Immunohistochemical Findings

In 27 cases of AML with inv(16), CYP2E1 was strongly positive in the cytoplasm of 20 (74.0%), focally/weakly expressed in five (18.5%), and negative in two (7.4%) cases. In contrast, in 10 cases of AMML without inv(16), CYP2E1 was strongly positive in one (10%), focally/weakly positive in three (30%), and negative in six (*p* < 0.001). We also assessed CYP2E1 expression in other types of AML (n = 22), CYP2E1 was clearly positive in seven (31.8%), focally/weakly in three (13.6%) and negative in 12 (54.6%), but the overall intensity of expression was significantly less than that of cases of AML with inv(16) (*p* < 0.001). CYP2E1 was negative in two cases of CMML, and in two normal BM controls. The staining pattern and positivity in AML with inv(16), AMML without inv(16) and other types of AML are demonstrated in Figure 3 and the data are summarized in Table 3.

**Table 3:** Immunohistochemical CYP2E1 protein expression in AML with inv(16), AMML without inv(16) and other types of AML.

Category	AML with inv(16) (n = 27)	AMML without inv(16) * (n = 10)	Other types of AML * (n = 22)
Strong	20	1	7
Weak/Focal	5	3	3
Negative	2	6	12

* *p*-*value <* 0.001.

**Figure 4:**
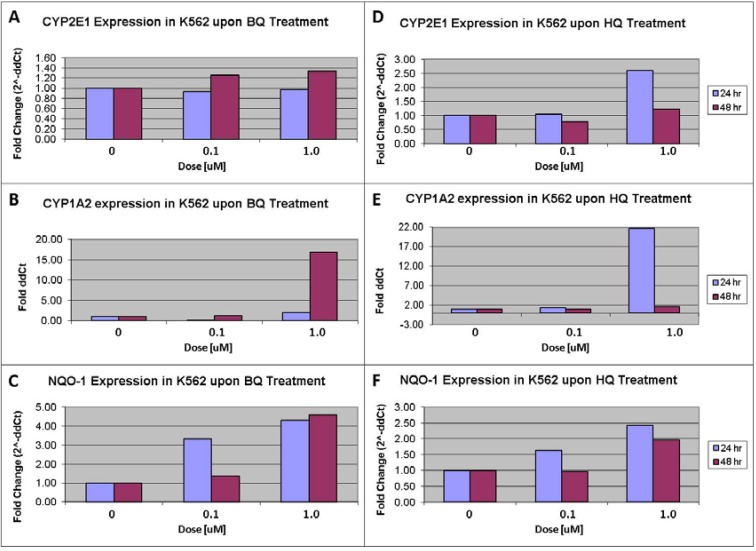
Representative mRNA expression levels of CYP2E1 (**A**); CYP1A2 (**B**) and NQO1 (**C**), as measured by real time reverse transcriptase-polymerase chain reaction in the K562 cell line, at 24 and 48 hours, after exposure to benzoquinone (BQ). Representative mRNA expression levels of CYP2E1 (**D**); CYP1A2 (**E**) and NQO1 (**F**) under the above conditions after exposure to HQ. Induction of CYP1A2 is marked (**B,E**); with mild induction in CYP2E1 (**A,D**) and NQO1 (**C,F**).

### 3.4. Cell Culture, Treatment and Quantitative Real-Time RT-PCR Assay

K562 cells were exposed to benzene analogues/metabolites 24 hours after plating. Benzene analogues/metabolites included the active benzoquinone/BQ) and inactive (hydroquinone/HQ). RNA expression levels of CYP2E1, CYP1A2 and NQO1 were measured at two different time points, 24 and 48 hours after exposure. As shown in Figure 4, exposure to the active form (BQ) of benzene induced elevated levels of CYP2E1, CYP1A2 and NQO1. Similarly, exposure to HQ induced elevated levels of CYP2E1, CYP1A2 and NQO1. Furthermore, exposure resulted in significantly higher levels of CYP1A2 compared with either CYP2E1 or NQO1 in these cell lines. 

## 4. Discussion

We report the results of CYP2E1 mRNA and protein analysis in BM samples from 11 patients with AML with inv(16). We found that CYP2E1 mRNA and protein are over-expressed in cases of AML with inv(16) compared with other types of AML and AMML without inv(16). Interestingly, we also found proportionally elevated NQO1 mRNA transcript levels in AML with inv(16). 

Using gene expression profiling, we showed earlier that the CYP2E1 gene is aberrantly over-expressed in cases of AML with inv(16) [16]. In this study, we confirmed this finding and demonstrated elevated levels of CYP2E1 mRNA and protein in cases of AML with inv(16). We also assessed CYP2E1 protein expression in other subtypes of AMLs, and found that elevated CYP2E1 expression was much more common in AML with inv(16) than in other AML types. Furthermore, we show that elevated expression of CYP2E1 was not a part of a generalized increase in cytochrome P450, as CYP1A2 was decreased. 

This study provides corroborating evidence implicating benzene in the pathogenesis of AML with inv(16), confirming the findings of others [5]. High levels of cytochrome p450 metabolizing enzymes have also been shown in AML with t(8;21)(q22;q22)/RUNX1-RUNX1T1. In the latter, AML resulted due to the formation of carcinogen benzopyrene-DNA adducts as a result of metabolism of environmental carcinogens by increased CYP1A1 [22]. Thus, for the development of AML, in addition to genetic abnormalities, environmental influences appear to be critical. One can further hypothesize that these carcinogens may have a role as secondary environmental triggers for leukemogenesis in the general population. 

The strong association between CYP2E1 and AML with inv(16) has implications for pathogenesis. First, this association allows one to infer that AML with inv(16) may be related to exposure to environmental toxins. The elevated expression of CYP2E1 alone would enhance the carcinogenic potential of environmental toxins. The general population is exposed to benzene and its toxic metabolites through gasoline, cigarette smoking, air pollution and dietary intestinal breakdown of proteins [23]. Elevated expression of CYP2E1 would increase the production of benzene metabolites including phenol, hydroquinone, catechol, and trihydroxybenzene. These metabolites induce toxic damage to DNA. Breaks in double stranded DNA compounded by inhibition of DNA repair can lead to chromosomal translocations such as inversion (16). [24,25]. In support of this idea, in addition to inv(16), other translocations such as alterations in chromosomes 5 and 7 also have shown to be associated with xenobiotic metabolites [13]. Second, it may be possible that targeted inhibition of CYP2E1 could play a role in preventing development of secondary malignancies. Pharmacological treatments to modify the levels of phase-I and phase-II enzymes already have been developed [26]. 

The explanation for the elevated levels of CYP2E1 specifically in most cases of AML with inv(16) remains unclear. AML with inv(16) is associated with a favorable outcome with long periods of complete remission [17,18]. While elevated CYP2E1 levels alone could lead to greater DNA toxicity, the metabolites of CYP2E1 are further processed by the phase-II enzyme NQO1. Thus, the functional activity of NQO1 could modify the overall effect of xenotoxin-induced damage. We report that patients with AML with inv(16) also have elevated levels of the NQO1 mRNA. The production of phase-I metabolites from increased levels of the CYP2E1 may lead to elevated levels of NQO1, as it known to be highly inducible by azo dyes and polycyclic aromatic hydrocarbons [27]. Increased NQO1 levels could serve to counteract the toxic damage induced by these metabolites, thus conferring a relatively favorable prognosis in AML with inv(16). 

There are limitations to this study, the most important being the lack of NQO1 protein levels to reinforce the mRNA levels in the myeloid system. The results, therefore, need to be confirmed in a larger subset of patients with well characterized polymorphisms in the CYP2E1 and NQO1 genes. Since the *NQO1* gene is located on chromosome 16q22.1, some authors have proposed that inversion (16) may be a primary event leading to disruption of this gene, thereby increasing the likelihood of leukemia [5]. Further studies to quantify protein expression and direct measurement of functional activity of CYP2E1 and NQO1 are needed to support the hypothesis we have proposed. 

The results from our *in vitro* experiments also provide direct evidence of induction of cytochrome p450 and NQO1 by benzene and its analogues/metabolites. Based on this data, we suggest that cases of AML, especially those types associated with recurrent genetic abnormalities, are uniquely associated with an increase in specific types of cytochrome p450 enzymes. This increase appears to be more than just a generalized increase in cytochrome p450 enzyme expression. The increase in specific types of cytochrome enzyme also appears to be unique to each type of leukemia as shown by others, such as specific induction of CYP1A1 in AML with t(8;21) [22] as well as CYP2E1 and NQO1 in AML with inv(16) [5,16]. It also has been shown that these xenotoxins seem to have a predilection to specifically cause AML associated with inv(16) [5], and high CYP2E1 levels have been shown in cases of AML with inv(16). 

## 5. Conclusions

In conclusion, we have shown simultaneously increased mRNA expression levels of the benzene metabolizing enzymes, CYP2E1 and NQO1, in AML with inv(16). Elevated levels of CYP2E1 mRNA and protein occur more commonly in AML with inv(16) compared with other types of AML. We further suggest that the balance between CYP2E1 (toxin producing) and NQO1 (detoxifying) enzymes may determines the overall carcinogenic potential of the xenotoxins. 
